# Navigating the obesity paradox in bladder cancer prognosis—insights from the Taiwan National Health Insurance System Database

**DOI:** 10.3389/fnut.2024.1433632

**Published:** 2024-12-11

**Authors:** Wen-Hsin Tseng, Ting-Yi Chiang, Chung-Han Ho, Steven K. Huang, Allen W. Chiu, Chien-Feng Li, Yow-Ling Shiue

**Affiliations:** ^1^Institute of Biomedical Science, National Sun Yat-sen University, Kaohsiung, Taiwan; ^2^Division of Urology, Department of Surgery, Chi Mei Medical Center, Tainan, Taiwan; ^3^Department of Medical Research, Chi Mei Medical Center, Yongkang, Tainan, Taiwan; ^4^Department of Information Management, Southern Taiwan University of Science and Technology, Tainan, Taiwan; ^5^Department of Urology, Shin Kong Wu Ho-Su Memorial Hospital, Taipei, Taiwan; ^6^Department of Pathology, Chi Mei Medical Center, Tainan, Taiwan; ^7^Institute of Precision Medicine, National Sun Yat-sen University, Kaohsiung, Taiwan

**Keywords:** bladder cancer, obesity paradox, Taiwan Cancer Registry, mortality, body mass index

## Abstract

**Purpose:**

This study investigates the complex relationship between body mass index (BMI) and bladder cancer outcomes, utilizing Taiwan’s national database. Bladder cancer remains a significant health concern, especially in Taiwan, prompting a comprehensive retrospective analysis to explore the impact of obesity on survival outcomes.

**Materials and methods:**

A meticulous exclusion process, based on Taiwan National Health Insurance System Database, refined the initial dataset of 15,086 bladder cancer patients to 10,352. Categorizing patients into BMI groups (underweight, normal weight, and obesity), the study examined baseline characteristics, comorbidities, and survival outcomes. The analysis involved Cox regression and subgroup assessments stratified by clinical stage.

**Results:**

Among our patients, 71.5% are male, 78.5% are over 60 years of age, and 18.8% are between 45 and 60 years old. Despite a higher prevalence of comorbidities, obesity patients exhibited a more favorable prognosis, supporting the obesity paradox. The overall and specific mortality ratio of obesity patients were 0.76 fold and 0.82-fold compared with normal-weight patients (overall: 95% confidence interval [CI], 0.71–0.82, *p* < 0.0001; specific: 95% CI, 0.75–0.90, p < 0.0001). Conversely, underweight patients displayed an increased risk of both overall and cancer-specific mortality compared to normal-weight patients (*p* < 0.0001).

**Conclusion:**

This study highlights the potential protective role of higher BMI in bladder cancer survival, revealing a more favorable prognosis among obesity patients, highlighting the need for cautious interpretation and suggesting avenues for future research. These insights could guide BMI-targeted intervention strategies, allowing clinicians to consider BMI as a factor in personalized treatment planning for bladder cancer patients.

## Introduction

Cancer treatment has evolved significantly from surgery and radiotherapy to targeted therapies and immunotherapies, achieving increased efficacy, reduced toxicity, and improved patient outcomes (1). Bladder cancer ranks among the prevalent malignancies affecting the urinary system globally, with an annual Age Standardized Incidence Rate (ASR) of 9.6 per 100,000 for males and 2.4 per 100,000 for females worldwide (2). Non-muscle-invasive bladder cancer is associated with an overall survival rate of approximately 90% (3). In patients with muscle-invasive bladder cancer undergoing bladder-preserving combined-modality therapy, the 5- and 10-year overall survival rates are 57 and 36%, respectively (4). In contrast, survival for metastatic bladder cancer remains poor, with a median survival of 3–6 months without treatment and extending to 12–15 months with treatment (5).

Data from the Taiwan Cancer Registry (TCR) shows that the projected age-standardized incidence rates per 100,000 people for men are 13.0 in 2020 and 10.4 in 2025, while for women, the rates are 4.7 in 2020 and 3.7 in 2025 (6). The accelerated aging of the population in Taiwan underscores the continued significance of bladder cancer as a serious health concern. Therefore, a comprehensive examination of potential risk factors and factors influencing progression remains imperative.

Overweight and obesity may exhibit an escalated risk of bladder cancer, demonstrating a Dose–Response correlation (7). This association is likely attributed to inflammatory processes, alterations in sex hormone metabolism, abnormal insulin and insulin-like growth factor levels, adipokine pathways, and microenvironment perturbations that contribute to tumor cell growth and proliferation (8). The implications for survival outcomes are intricate, marked by contradictory findings in the literature. Some studies report a negative correlation between higher body mass index (BMI) and prognosis; for instance, a large multi-institutional series showed that obesity is associated with worse cancer-specific outcomes in patients treated with radical cystectomy for urothelial carcinoma of the bladder, with higher risks of disease recurrence and cancer-specific mortality (9). However, other studies identify better survival outcomes. Data from the PROspective MulticEnTer RadIcal Cystectomy Series (2011) indicated that the overall survival rate of obese patients was superior to that of normal-weight patients (10). Additionally, for patients with non-muscle invasive bladder cancer treated with transurethral resection of bladder tumor and adjuvant intravesical BCG, Huang et al. (11) found that obese patients had better overall survival than non-obese patients. This phenomenon, known as the “obesity paradox,” refers to the observation that higher BMI is associated with decreased mortality risk in certain cancers, contradicting expectations (12). The obesity paradox is well recognized in the cardio-metabolic literature but is less commonly discussed in oncology (13). However, it has been reported in various cancers, including lymphoma, leukemia, colorectal, gastric, renal, and lung cancers (14).

Given that Asian populations typically exhibit higher body fat accumulation and elevated levels of adipocytokines at equivalent BMI levels compared to their Western counterparts (15), assessing this correlation within the Taiwanese group is paramount. The primary objective of this study is to rigorously analyze the association between BMI and bladder cancer, utilizing nationally representative data from the Taiwanese population and stratifying the analysis by cancer stages. This approach aims to contribute valuable insights into the complex interplay between adiposity and bladder cancer risk and prognosis.

## Materials and methods

### Data source

Datasets from the TCR, the Taiwan’s National Health Insurance Research Database (NHIRD), and the Taiwan’s Cause-of-Death Database were used in this study. These claims datasets were all from the Health and Welfare Data Science Center (HWDC), an integrated health-related database center. The TCR, established in 1979, monitors cancer incidence and mortality rates across Taiwan. It collects data on individual demographics, cancer stages, primary tumor sites, histology, and treatment types. Bladder cancer incidence data from 1997 to 2016 were obtained from this publicly available, nationwide, population-based registry. Since reaching maturity in 2003, the registry has maintained high data quality, with timeliness under 14 months, completeness exceeding 98%, a morphological verification rate of approximately 93%, and fewer than 1% of cases registered solely by death certificate (6). NHIRD is based on Taiwan’s national health insurance program, which includes detailed healthcare information of more than 99% of Taiwan’s population from 1996 to 2018. For research purposes, the HWDC released the de-identified claims to the public in an anonymous format. This study was conducted in compliance with the Declaration of Helsinki and has been approved by the Research Ethics Committee of Chi Mei Hospital (IRB no. 11301–017). In addition, patient informed consent was waived by the Research Ethics Committee of Chi Mei Hospital.

### Definitions of study subjects

Patients with bladder cancer between 2011 and 2018 were selected from the TCR using the International Classification of Diseases for Oncology, 3rd edition (ICD-O-3): C67. The patients with incomplete information in the TCR were excluded from this study. Exclusion criteria were applied to ensure a homogeneous sample and reduce bias. Patients with clinical stage 0 or missing data were excluded due to the impact of unclear staging on prognosis and treatment analysis. Those with missing height or weight data were excluded to maintain accurate BMI calculations, a key factor in the study. Patients without data on smoking, drinking, or betel nut chewing were excluded as these lifestyle factors can influence cancer outcomes, and missing data would introduce confounding. Finally, patients under 20 years old were excluded due to the extreme rarity of cancer in children and adolescents (16), with available data primarily limited to case reports, ensuring a more clinically relevant and homogenous study population.

**Figure 1:**
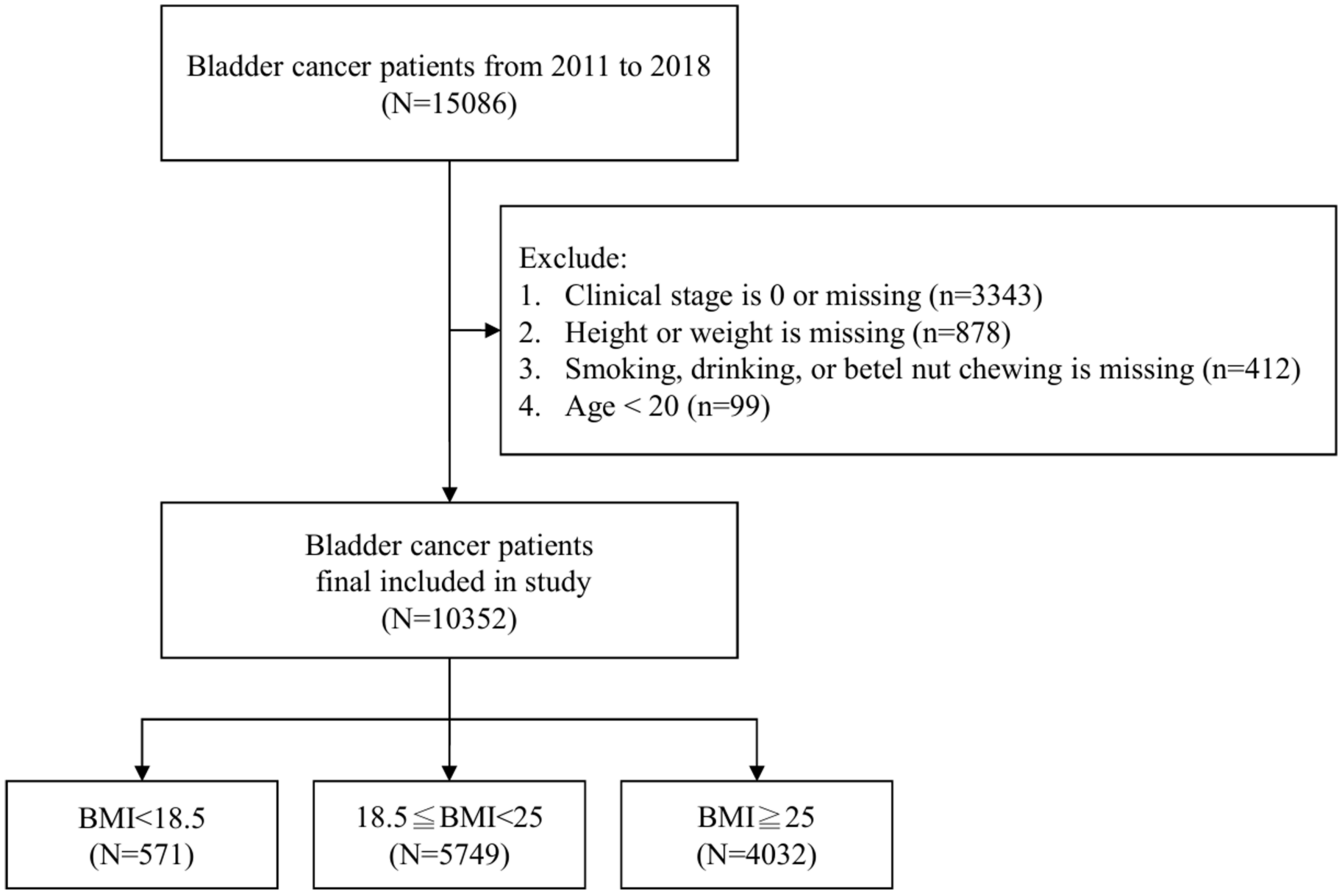
Exclusion process in the initial dataset of 15,086 bladder cancer patients, resulting in 10,352 individuals categorized into underweight (BMI < 18.5, *n* = 571), normal weight (18.5 ≤ BMI < 25, *n* = 5,749), and overweight (BMI ≥ 25, *n* = 4,032).

The World Health Organization (WHO) defines overweight as a BMI ≥25 kg/m^2^ and obesity as a BMI ≥30 kg/m^2^, regardless of race or sex (17). However, Asian populations have an increased risk of diabetes and cardiovascular diseases even at a BMI ≤25 kg/m^2^. Consequently, the WHO Asia-Pacific region defines overweight as BMI ≥23 kg/m^2^ and obesity as BMI ≥25 kg/m^2^ (18). Therefore, in our study, we used a BMI cutoff of 25 kg/m^2^ to define obesity. A flowchart summarizing the selection of study subjects is presented in Figure 1.

### Measurement

The primary outcome of this study was mortality. Mortality was determined from the Cause-of-Death Dataset. The mortality risk was estimated using two endpoints: overall and cancer-specific mortalities. The time for the overall mortality was set from the date of bladder cancer diagnosis to the date of death, regardless of the cause. Cancer-specific mortality was defined as the cause of death due to bladder cancer.

Patients with comorbidities were defined as patients with the diseases included below at least 1 year before the diagnosis of bladder cancer. This definition was based on the International Classification of Diseases, Ninth Revision, Clinical Modification (ICD-9-CM) codes in Appendix 1. Comorbidities included the following: myocardial infarction; congestive heart failure, peripheral vascular disease, cardiovascular disease, dementia, chronic obstructive pulmonary disease, renal disease, diabetes mellitus, hypertension, and liver disease.

In the stratification, patients with bladder cancer were categorized by gender, age group, and clinical stage to assess the risk of overall and cancer-specific mortality across different BMI categories. This approach allowed for a more nuanced understanding of how these factors influence mortality risks. Gender plays a significant role in bladder cancer outcomes, as men have a fourfold higher incidence rate than women, while female patients often present with higher-grade disease and tend to experience worse outcomes (19). Age was divided into three categories: 20–45 years, 45–60 years, and 60 years or older. Lastly, patients were stratified by clinical stage, which was divided into early-stage (Stage I and II) and advanced-stage (Stage III and IV) bladder cancer. Clinical stage is a critical determinant of survival, with higher stages indicating more aggressive disease. Stratifying by clinical stage helps control for disease severity, ensuring accurate and meaningful comparisons between groups.

### Statistical analysis

Continuous variables are presented as means ± standard deviations, while categorical variables are presented as frequencies with percentages. The Cox proportional regression model was chosen for its ability to assess the relationship between predictors and the time to an event, and was used to estimate the hazard ratios (HRs) of the overall and cancer-specific mortalities. This model is ideal for survival analysis, as it does not assume a specific distribution for the time variable. Confounders like age, sex, smoking status, and comorbidities were adjusted for to minimize bias and clarify the relationship between the main exposure and outcome. Adjusted HRs were calculated from the multivariable Cox regression with adjustment of age at diagnosis, sex, body mass index (BMI), Charlson Comorbidity Index (CCI) score, drinking, chewing betel nut, clinical stage, treatment type, and comorbidities. The stratified analysis was also presented to estimate the risk ratios of the overall and cancer-specific mortalities between bladder cancer patients with underweight, normal-weight and overweight patients. To avoid violation of the proportional hazards assumption, the estimated HRs were checked using the Schoenfeld residuals test. In addition, considering the differences in BMI classification between Asian populations and WHO standards, a sensitivity analysis was conducted using Taiwan’s BMI classification (underweight: <18.5 kg/m^2^; normal weight: 18.5–24 kg/m^2^; overweight: ≥24 kg/m^2^) to compared with the findings from WHO classification. The SAS 9.4 (SAS Institute, Inc., Cary, NC, United States) was used to perform all statistical analyses. A *p*-value <0.05 was set for statistical significance.

## Results

In the initial dataset of 15,086 patients, a meticulous exclusion process led to the removal of 4,732 individuals from the analysis, following criteria such as clinical stage 0 or missing data (*n* = 3,343), absence of information on weight or height (*n* = 878), unrecorded data regarding smoking, drinking, or betel nut chewing (*n* = 412), and an age less than 20 (*n* = 99). The refined sample of 10,352 bladder cancer patients was then categorized into three distinct BMI groups: underweight (BMI < 18.5, *n* = 571), normal weight (18.5 ≤ BMI < 25, *n* = 5,749), and overweight (BMI ≥ 25, *n* = 4,032) as illustrated in Figure 1.

The baseline demographic and disease characteristics of bladder cancer patients, stratified by BMI category, revealed significant differences. Patients in the higher BMI cohort were younger, predominantly male (*p* < 0.0001) compared to those in lower BMI categories. Overweight patients had a higher prevalence of comorbidities, including diabetes (30.68%), hyperlipidemia (27.31%), and hypertension (57.19%), surpassing their normal or underweight counterparts (*p* < 0.0001). Overweight patients also exhibited a more favorable prognosis, marked by a lower overall mortality (27.48%), reduced bladder cancer-specific mortality (18.08%), and an extended survival period (1.78 ± 1.42 years) (*p* < 0.0001) (Table 1).

**Table 1:** The characteristics among bladder cancer patients in different BMI groups.

	BMI < 18.5 (*N* = 571)	18.5 ≦ BMI < 25 (*N* = 5,749)	BMI ≧ 25 (*N* = 4,032)	*p*-value
Age	73.47 ± 13.48	70.29 ± 12.39	67.64 ± 11.93	<0.0001
Age group				
20–45	15 (2.63)	139 (2.42)	126 (3.13)	<0.0001
45–60	83 (14.54)	982 (17.08)	876 (21.73)	
≧60	473 (82.84)	4,628 (80.50)	3,030 (75.15)	
Gender				
Male	326 (57.09)	4,061 (70.64)	3,013 (74.73)	<0.0001
Female	245 (42.91)	1,688 (29.36)	1,019 (25.27)	
Clinical stage				
I	212 (37.13)	2,893 (50.32)	2,162 (53.62)	<0.0001
II	161 (28.20)	1,418 (24.67)	1,038 (25.74)	
III	93 (16.29)	763 (13.27)	446 (11.06)	
IV	105 (18.39)	675 (11.74)	386 (9.57)	
Smoking				
Never	435 (76.18)	3,923 (68.24)	2,658 (65.92)	<0.0001
Ever/current	136 (23.82)	1826 (31.76)	1,374 (34.08)	
Drinking				
Never	508 (88.97)	4,755 (82.71)	3,220 (79.86)	<0.0001
Ever/current	63 (11.03)	994 (17.29)	812 (20.14)	
Betel nut chewing				
Never	554 (97.02)	5,412 (94.14)	3,704 (91.87)	<0.0001
Ever/current	17 (2.98)	337 (5.86)	328 (8.13)	
CCI score	2.75 ± 2.62	2.22 ± 2.25	2.16 ± 2.17	<0.0001
CCI group				
0	131 (22.94)	1,643 (28.58)	1,146 (28.42)	0.0029
1–2	198 (34.68)	2059 (35.81)	1,494 (37.05)	
> =3	242 (42.38)	2047 (35.61)	1,392 (34.52)	
Comorbidity				
Myocardial infarct	9 (1.58)	74 (1.29)	58 (1.44)	0.7371
Congestive heart failure	42 (7.36)	265 (4.61)	215 (5.33)	0.0094
Peripheral vascular disease	17 (2.98)	128 (2.23)	79 (1.96)	0.2608
Cerebrovascular disease	68 (11.91)	540 (9.39)	354 (8.78)	0.0507
Dementia	39 (6.83)	219 (3.81)	91 (2.26)	<0.0001
Chronic pulmonary disease	96 (16.81)	522 (9.08)	376 (9.33)	<0.0001
Connective tissue disease	5 (0.88)	93 (1.62)	46 (1.14)	0.0782
Ulcer disease	75 (13.13)	591 (10.28)	378 (9.38)	0.0155
Mild liver disease	25 (4.38)	399 (6.94)	265 (6.57)	0.0620
Diabetes without end organ damage	70 (12.26)	1,140 (19.83)	1,137 (28.20)	<0.0001
Hemiplegia	5 (0.88)	38 (0.66)	37 (0.92)	0.3465
Moderate or severe renal disease	151 (26.44)	1,036 (18.02)	543 (13.47)	<0.0001
Diabetes with end organ damage	36 (6.30)	404 (7.03)	364 (9.03)	0.0005
Moderate or severe liver disease	3 (0.53)	18 (0.31)	12 (0.30)	0.6604
Diabetes	84 (14.71)	1,279 (22.25)	1,237 (30.68)	<0.0001
Hyperlipidemia	59 (10.33)	1,100 (19.13)	1,101 (27.31)	<0.0001
Hypertension	233 (40.81)	2,690 (46.79)	2,306 (57.19)	<0.0001
Treatment				
Operation	504 (88.27)	5,477 (95.27)	3,906 (96.88)	<0.0001
Radiotherapy	104 (18.21)	808 (14.05)	492 (12.20)	0.0001
Chemotherapy	107 (18.74)	1,203 (20.93)	838 (20.78)	0.4690
Time to follow up	1.96 ± 2.01	2.65 ± 2.14	2.87 ± 2.12	<0.0001
Death	330 (57.79)	2,131 (37.07)	1,108 (27.48)	<0.0001
Time to mortality	1.11 ± 1.17	1.64 ± 1.54	1.78 ± 1.42	<0.0001
Death in BC	206 (36.08)	1,321 (22.98)	729 (18.08)	<0.0001

In terms of mortality risk, adjusted Cox regression analyses showed that the overall mortality rate in overweight patients was 0.76-fold (95% confidence interval [CI], 0.71–0.82, *p* < 0.0001) adjusted HR compared with normal-weight patients. In addition, the specific mortality rate of overweight patients was 0.82-fold (95% CI, 0.75–0.90, *p* < 0.0001) adjusted HR compared with normal-weight patients. The HRs with confidence intervals provide an estimate of the relative risk of overall and cancer-specific mortality. The crude HRs reflect the unadjusted risk, while the adjusted HRs (AHRs) account for factors such as age, sex, comorbidities, and treatment modalities, offering a more accurate assessment of the impact of each factor on survival outcomes. Conversely, the overall and specific mortality ratio of underweight patients were 1.58 fold and 1.50-fold compared with normal-weight patients (overall: 95% confidence interval [CI], 1.41–1.78, *p* < 0.0001; specific: 95% CI, 1.29–1.74, *p* < 0.0001) (Table 2).

**Table 2:** The risk of overall and cancer-specific mortality among bladder cancer patients and stratified by gender, age group, and clinical stage.

	Overall mortality						Cancer-specific mortality				
	Patients	Death (%)	Crude HR	*p*-value	AHR	*p*-value	Death (%)	Crude HR	*p*-value	AHR	*p*-value
BMI											
BMI < 18.5	571	330 (57.79)	2.04 (1.81–2.29)	<0.0001	1.58 (1.41–1.78)	<0.0001	206 (36.08)	2.02 (1.75–2.35)	<0.0001	1.50 (1.29–1.74)	<0.0001
18.5≦BMI < 25	5,749	2,131 (37.07)	Ref.		Ref.		1,321 (22.98)	Ref.		Ref.	
BMI≧25	4,032	1,108 (27.48)	0.69 (0.64–0.74)	<0.0001	0.76 (0.71–0.82)	<0.0001	729 (18.08)	0.73 (0.67–0.80)	<0.0001	0.82 (0.75–0.90)	<0.0001
Gender											
Male	7,440	2,465 (33.13)	0.86 (0.80–0.92)	<0.0001	0.94 (0.87–1.02)	0.1407	1,540 (20.7)	0.82 (0.75–0.90)	<0.0001	0.91 (0.83–1.00)	0.0612
Female	2,952	1,101 (37.3)	Ref.		Ref.		716 (24.25)	Ref.		Ref.	
Clinical stage											
I	5,267	1,089 (20.68)	Ref.		Ref.		450 (8.54)	Ref.		Ref.	
II	2,617	970 (37.07)	2.14 (1.96–2.33)	<0.0001	2.13 (1.95–2.33)	<0.0001	655 (25.03)	3.45 (3.06–3.89)	<0.0001	3.35 (2.96–3.79)	<0.0001
III	1,302	621 (47.7)	3.53 (3.20–3.89)	<0.0001	3.62 (3.26–4.03)	<0.0001	457 (35.1)	6.12 (5.37–6.97)	<0.0001	6.02 (5.24–6.91)	<0.0001
IV	1,166	889 (76.24)	8.03 (7.34–8.79)	<0.0001	8.14 (7.33–9.04)	<0.0001	694 (59.52)	14.46 (12.82–16.31)	<0.0001	13.88 (12.11–15.91)	<0.0001
Age group											
20–45	280	50 (17.86)	Ref.		Ref.		44 (15.71)	Ref.		Ref.	
45–60	1941	388 (19.99)	1.15 (0.85–1.54)	0.3640	1.09 (0.81–1.46)	0.5773	283 (14.58)	0.94 (0.69–1.30)	0.7208	0.89 (0.64–1.22)	0.4517
≧60	8,131	3,131 (38.51)	2.79 (2.11–3.68)	<0.0001	2.22 (1.67–2.94)	<0.0001	1929 (23.72)	1.89 (1.40–2.54)	<0.0001	1.53 (1.13–2.07)	0.0058
Smoking											
Never	7,016	2,456 (35.01)	Ref.		Ref.		1,574 (22.43)	Ref.		Ref.	
Ever/current	3,336	1,113 (33.36)	0.91 (0.84–0.97)	0.0056	1.12 (1.03–1.22)	0.0100	682 (20.44)	0.87 (0.79–0.95)	0.0021	1.04 (0.93–1.16)	0.4643
Drinking											
Never	8,483	2,984 (35.18)	Ref.		Ref.		1886 (22.23)	Ref.		Ref.	
Ever/current	1869	585 (31.3)	0.82 (0.75–0.89)	<0.0001	0.90 (0.81–1.00)	0.0510	370 (19.8)	0.82 (0.74–0.92)	0.0006	0.93 (0.81–1.06)	0.2613
Betel nut chewing											
Never	9,670	3,350 (34.64)	Ref.		Ref.		2,119 (21.91)	Ref.		Ref.	
Ever/current	682	219 (32.11)	0.90 (0.79–1.03)	0.1355	1.06 (0.91–1.23)	0.4547	137 (20.09)	0.89 (0.75–1.06)	0.1880	1.04 (0.86–1.26)	0.6969
CCI group											
0	2,920	774 (26.51)	Ref.		Ref.		557 (19.08)	Ref.		Ref.	
1–2	3,751	1,151 (30.69)	1.19 (1.09–1.30)	0.0002	1.29 (1.17–1.41)	<0.0001	734 (19.57)	1.05 (0.94–1.17)	0.3861	1.20 (1.07–1.35)	0.0014
≧3	3,681	1,644 (44.66)	2.09 (1.92–2.28)	<0.0001	2.09 (1.90–2.30)	<0.0001	965 (26.22)	1.67 (1.51–1.86)	<0.0001	1.80 (1.60–2.03)	<0.0001
Treatment											
Operation	9,887	3,216 (32.53)	0.24 (0.22–0.27)	<0.0001	0.54 (0.48–0.60)	<0.0001	2008 (20.31)	0.22 (0.19–0.25)	<0.0001	0.56 (0.49–0.65)	<0.0001
Radiotherapy	1,404	750 (53.42)	2.10 (1.94–2.28)	<0.0001	1.08 (0.99–1.18)	0.0768	579 (41.24)	2.66 (2.42–2.92)	<0.0001	1.19 (1.08–1.32)	0.0007
Chemotherapy	2,148	978 (45.53)	1.66 (1.54–1.79)	<0.0001	0.79 (0.73–0.86)	<0.0001	755 (35.15)	2.18 (2.00–2.38)	<0.0001	0.84 (0.76–0.93)	0.0006
Comorbidity											
Diabetes	2,600	1,009 (38.81)	1.29 (1.20–1.38)	<0.0001	1.00 (0.92–1.08)	0.9527	594 (22.85)	1.15 (1.05–1.27)	0.0028	0.97 (0.87–1.08)	0.5169
Hyperlipidemia	2,260	664 (29.38)	0.82 (0.75–0.89)	<0.0001	0.75 (0.68–0.82)	<0.0001	421 (18.63)	0.82 (0.74–0.91)	0.0002	0.80 (0.71–0.89)	<0.0001
Hypertension	5,229	1965 (37.58)	1.30 (1.21–1.39)	<0.0001	1.11 (1.03–1.19)	0.0044	1,183 (22.62)	1.15 (1.06–1.25)	0.0007	1.04 (0.95–1.13)	0.4403

In the subgroup analysis, overweight patients consistently exhibited a lower mortality rate, a trend that was significant across both genders and in overall and cancer-specific mortality (*p* < 0.01). When stratified by clinical stage, underweight patients in stages I, II, and III showed an increased risk of both overall and cancer-specific mortality (*p* < 0.01). Although a higher mortality rate was observed in stage IV underweight patients, this did not reach statistical significance (*p* > 0.05). Overweight patients exhibited a significantly lower overall and cancer-specific mortality rate across all stages, with the exception of cancer-specific mortality in stage I patients (AHR: 0.92, 95% CI: 0.75–1.12, *p* = 0.4104) (Table 3).

**Table 3:** The risk of overall and cancer-specific mortality among bladder cancer patients in different BMI groups stratified by gender, age group, and clinical stage.

	Overall mortality				Cancer-specific mortality		
	Patients	Death (%)	AHR	*p*-value	Death (%)	AHR	*p*-value
Overall							
BMI < 18.5	571	330 (57.79)	1.58 (1.41–1.78)	<0.0001	206 (36.08)	1.50 (1.29–1.74)	<0.0001
18.5 ≦ BMI < 25	5,749	2,131 (37.07)	Ref.		1,321 (22.98)	Ref.	
BMI ≧ 25	4,032	1,108 (27.48)	0.76 (0.71–0.82)	<0.0001	729 (18.08)	0.82 (0.75–0.90)	<0.0001
Stratified							
Male							
BMI < 18.5	326	203 (62.27)	1.69 (1.45–1.96)	<0.0001	121 (37.12)	1.52 (1.25–1.84)	<0.0001
18.5 ≦ BMI < 25	4,061	1,499 (36.91)	Ref.		925 (22.78)	Ref.	
BMI ≧ 25	3,013	763 (25.32)	0.73 (0.67–0.80)	<0.0001	494 (16.4)	0.79 (0.71–0.89)	<0.0001
Female							
BMI < 18.5	245	127 (51.84)	1.48 (1.22–1.80)	<0.0001	85 (34.69)	1.52 (1.19–1.93)	0.0006
18.5 ≦ BMI < 25	1,688	632 (37.44)	Ref.		396 (23.46)	Ref.	
BMI ≧ 25	1,019	345 (33.86)	0.84 (0.73–0.96)	0.0100	235 (23.06)	0.90 (0.76–1.06)	0.2100
20–45							
BMI < 18.5	15	3 (20)	2.13 (0.56–8.14)	0.2683	3 (20)	2.54 (0.65–9.89)	0.1787
18.5 ≦ BMI < 25	139	27 (19.42)	Ref.		23 (16.55)	Ref.	
BMI ≧ 25	126	20 (15.87)	1.01 (0.50–2.02)	0.9884	18 (14.29)	1.05 (0.50–2.23)	0.8974
45–60							
BMI < 18.5	83	23 (27.71)	1.23 (0.79–1.91)	0.3600	18 (21.69)	1.36 (0.82–2.25)	0.2308
18.5 ≦ BMI < 25	982	223 (22.71)	Ref.		159 (16.19)	Ref.	
BMI ≧ 25	876	142 (16.21)	0.70 (0.56–0.87)	0.0011	106 (12.1)	1.04 (0.76–1.43)	0.7897
≧60							
BMI < 18.5	473	304 (64.27)	1.65 (1.45–1.86)	<0.0001	185 (39.11)	1.53 (1.30–1.79)	<0.0001
18.5 ≦ BMI < 25	4,628	1881 (40.64)	Ref.		1,139 (24.61)	Ref.	
BMI ≧ 25	3,030	946 (31.22)	0.76 (0.70–0.83)	<0.0001	605 (19.97)	0.83 (0.75–0.91)	0.0002
Stage I							
BMI < 18.5	212	87 (41.04)	2.15 (1.72–2.70)	<0.0001	39 (18.4)	2.48 (1.76–3.49)	<0.0001
18.5 ≦ BMI < 25	2,893	652 (22.54)	Ref.		243 (8.4)	Ref.	
BMI ≧ 25	2,162	350 (16.19)	0.69 (0.61–0.79)	<0.0001	168 (7.77)	0.92 (0.75–1.12)	0.4104
Stage II							
BMI < 18.5	161	90 (55.9)	1.74 (1.39–2.18)	<0.0001	57 (35.4)	1.64 (1.24–2.18)	0.0006
18.5 ≦ BMI < 25	1,418	562 (39.63)	Ref.		377 (26.59)	Ref.	
BMI ≧ 25	1,038	318 (30.64)	0.78 (0.68–0.89)	0.0004	221 (21.29)	0.80 (0.67–0.95)	0.0091
Stage III							
BMI < 18.5	93	62 (66.67)	1.67 (1.27–2.19)	0.0003	43 (46.24)	1.56 (1.12–2.16)	0.0081
18.5 ≦ BMI < 25	763	383 (50.2)	Ref.		284 (37.22)	Ref.	
BMI ≧ 25	446	176 (39.46)	0.73 (0.61–0.88)	0.0008	130 (29.15)	0.72 (0.58–0.89)	0.0028
Stage IV							
BMI < 18.5	105	91 (86.67)	1.15 (0.91–1.45)	0.2432	67 (63.81)	1.10 (0.84–1.43)	0.4989
18.5 ≦ BMI < 25	675	534 (79.11)	Ref.		417 (61.78)	Ref.	
BMI ≧ 25	386	264 (68.39)	0.84 (0.72–0.98)	0.0240	210 (54.4)	0.84 (0.71–1.00)	0.0507
NMIBC							
BMI < 18.5	212	87 (41.04)	2.15 (1.72–2.70)	<0.0001	39 (18.4)	2.48 (1.76–3.49)	<0.0001
18.5 ≦ BMI < 25	2,893	652 (22.54)	Ref.		243 (8.4)	Ref.	
BMI ≧ 25	2,162	350 (16.19)	0.69 (0.61–0.79)	<0.0001	168 (7.77)	0.92 (0.75–1.12)	0.4104
MIBC							
BMI < 18.5	359	243 (67.69)	1.53 (1.34–1.76)	<0.0001	167 (46.52)	1.45 (1.22–1.70)	<0.0001
18.5 ≦ BMI < 25	2,856	1,479 (51.79)	Ref.		1,078 (37.75)	Ref.	
BMI ≧ 25	1870	758 (40.53)	0.77 (0.70–0.84)	<0.0001	561 (30)	0.78 (0.70–0.86)	<0.0001

**Figure 2:**
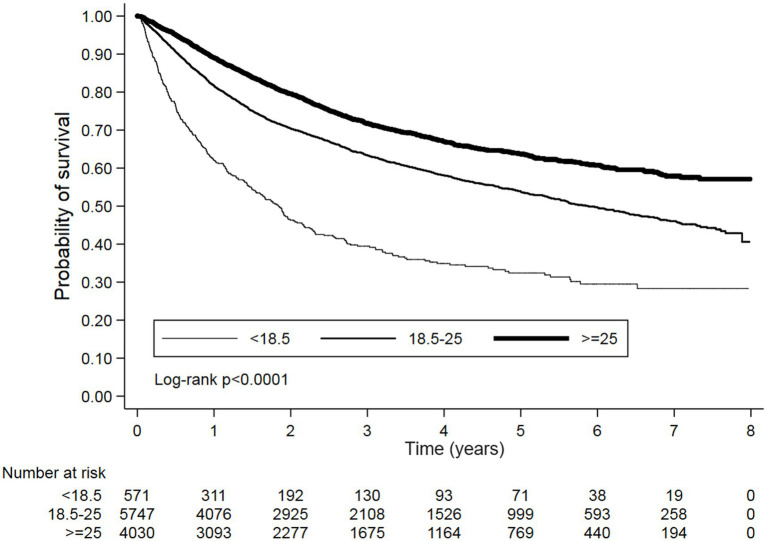
Kaplan–Meier survival curves for OS stratified by BMI categories. A significant difference in survival was observed between the groups (Log-rank *p* < 0.0001). The number of patients at risk is indicated at the bottom of the figure for each time point.

**Figure 3:**
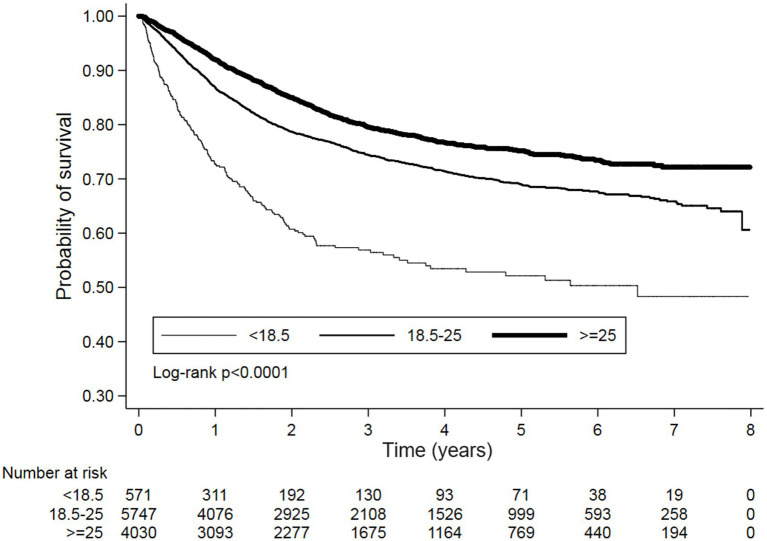
Kaplan–Meier survival curves for PFS stratified by BMI categories. A significant difference in survival was observed between the groups (Log-rank *p* < 0.0001). The number of patients at risk is indicated at the bottom of the figure for each time point.

Kaplan–Meier survival curves also demonstrated significantly trends of OS and PFS between different BMI categories with log-rank *p* < 0.0001 (Figures 2, 3). Additionally, the sensitivity analyses using Taiwan’s BMI classification also presented the consistent results with the above primary analysis (Supplementary Table S1).

## Discussion

This study represents the inaugural comprehensive retrospective analysis within Taiwan’s national database, exploring the survival outcomes of bladder cancer among patients categorized by different BMI groups. Within the overweight group, despite a higher prevalence of comorbidities, a noteworthy decrease in both overall and cancer-specific mortality was observed, accompanied by an extended survival period. Our data support the obesity paradox hypothesis for greater precision, suggesting that the higher prevalence of comorbidities does not appear to significantly impact overall mortality in the subsequent 2–3 years.

The relationship between obesity and bladder cancer is complex, with ongoing debate in the scientific community. Systematic reviews suggest a modest association between obesity and bladder cancer incidence (RR = 1.1, 95% CI 1.07–1.13) and a similar trend for overweight individuals (RR = 1.07, 95% CI 1.03–1.1). A dose–response analysis indicated a BMI increase of 5 kg/m^2^ raised bladder cancer risk by 3% (RR = 1.03, 95% CI 1.02–1.05) (20). Data from the Korean National Health Insurance database found the highest bladder cancer risk in those with BMI ≥ 30 (HR = 1.17) (21). Additionally, a meta-analysis revealed a significantly higher recurrence rate in obese patients (HR = 1.76, 95% CI: 1.36–2.28) and a linear relationship between BMI and recurrence risk (HR = 1.01, 95% CI: 1.01–1.02), though obesity was not significantly associated with overall survival (HR = 1.21, 95% CI: 0.97–1.52) (22).

To elucidate the complex relationship between obesity and bladder cancer, several mechanisms have been proposed. A study by Zhao et al. (23) demonstrated that elevated levels of IGF-1 were associated with increased bladder cancer risk, while higher levels of IGF-binding protein-3 were protective, with a dose–response relationship between these factors and cancer risk. In obesity, hyperinsulinemia caused by insulin resistance leads to reduced growth hormone secretion, but IGF-I levels remain stable due to increased hepatic GH sensitivity, with suppressed IGFBP-1 levels in response to elevated insulin (24), which may decreasing the protection toward bladder cancer. Another study in mice fed a high-fat diet revealed greater bladder inflammation and premalignant alterations, including increased dysplasia, proliferation, apoptosis, and activation of inflammatory pathways (NFκB, IKKβ, JNK, and c-JUN). The tumor microenvironment plays a key role in cancer development, growth, and progression (25).

The role of BMI in predicting survival outcomes for bladder cancer patients exhibits substantial variability across studies. A meta-analysis focusing on urothelial cancer patients undergoing radical surgery demonstrated that overweight individuals had improved Cancer-Specific Survival (CSS) and Recurrence-Free Survival (RFS), while obesity and underweight were associated with unfavorable survival outcomes (26). The significant heterogeneity observed in this review was partially attributed to the inclusion of studies from both Asian and Western populations, utilizing varying BMI category definitions. In our study, we addressed this heterogeneity by employing WHO-defined BMI categories tailored to the Asian population, aiming to rectify these discrepancies and reinforcing the observed findings associated with the obesity paradox. However, our database has limitations, lacking long-term follow-up data, which may be relevant to the study by Arthuso et al. (27), indicating diminishing differences observed in longer-term survival up to 5 years.

In our study, a higher proportion of obese patients were found to have diabetes, hypertension, and hyperlipidemia. Studies including meta-analyses have shown a positive correlation between these comorbidities and bladder cancer risk, as well as metabolic syndrome (28–30). A systematic review indicated that diabetic patients had a significantly higher risk for all-cause mortality (HR 1.24, 95% CI: 1.07–1.44) and cancer-specific mortality (HR 1.67, 95% CI: 1.29–2.16) compared to non-diabetics (31). Teleka et al. (32) found that systolic blood pressure was positively associated with bladder cancer-specific mortality (HR 1.10 [1.01–1.20]) among never-smokers, although weaker and non-significant associations were observed for others. Regarding hyperlipidemia, Tu et al. (33) reported that elevated cholesterol (CHOL), low HDL, and elevated triglycerides (TG) were linked to worse OS. Elevated CHOL, LDL, and TG, as well as lower HDL, significantly affected PFS. Furthermore, studies have shown that diet-induced and LDLR deficiency-induced hypercholesterolemia can enhance both bladder cancer stemness and progression (34). However, in our study, diabetes and hypertension did not show decreased OS or PFS, while hyperlipidemia was associated with significant OS and PFS benefits. This suggests that specific research on bladder cancer mortality in Taiwanese populations, and the impact of lipid profiles, warrants further investigation. Given the evidence of increased mortality in bladder cancer patients with comorbidities, and the fact that among 28 cancer types, bladder cancer patients have the highest risk of dying from cardiovascular disease (35), the persistence of the “obesity paradox” highlights its important impact on outcomes.

Although our study supports the “obesity paradox” hypothesis in bladder cancer, with observed reductions in both overall and cancer-specific mortality for overweight patients, these findings warrant cautious interpretation. The data contribute to the notion of the obesity paradox but highlight the need for greater precision in understanding how BMI influences bladder cancer outcomes. The obesity paradox has been observed in lung, renal, and metastatic melanoma cancers (36), which typically experience higher rates of surgical complications and early cancer-related deaths. Variations in BMI at pre-, peri-, and post-diagnosis may influence results (13), as evidenced by sarcopenia independently predicting Overall Survival (OS) and Cancer-Specific Survival (CSS) in a multicenter study of patients undergoing radical cystectomy for bladder cancer (37). Given that BMI is an inadequate measure of adiposity, other measurements, such as adipose compartment areas, may offer a more accurate assessment of the paradox. Studies adjusting for lean muscle wasting found no significant associations between obesity or adiposity measurements and all-cause mortality in patients treated with radical cystectomy (38). Confounding factors such as smoking, socioeconomic status, physical activity, diet, and ethnicity could impact our assumptions; for instance, exercise decreases and current smoking increases the risk of bladder cancer-specific mortality (39).

Additional biases, such as collider stratification bias, must be considered. Smoking, a well-established risk factor for bladder cancer, acts as a collider variable. Consequently, among non-obese cancer patients, the likelihood of other risk factors, such as smoking, increases, potentially generating an artificial inverse association (13). However, in our study, the obesity paradox persisted even after stratifying for smoking status, with underweight patients showing significantly increased overall mortality and overweight patients demonstrating significantly decreased overall mortality (Table 3).

Regarding detection bias, evidence indicates that diabetes and excess body weight can negatively impact bladder cancer prognosis and outcomes (31, 40). The phenomenon of reverse causality, where the prevalence of sarcopenia significantly increases during cancer treatment in patients with bladder cancer (41), further underscores the intricacies involved. These considerations emphasize the necessity for a cautious interpretation of our findings, acknowledging the potential influence of these biases on our understanding of the relationship between BMI and bladder cancer outcomes.

In the subgroup analysis of our study, while the obesity paradox persisted across stages, some exceptions were noted. Stage IV underweight patients displayed an insignificant mortality rate, and overweight stage I patients exhibited higher cancer-specific mortality. These findings align with previous studies, suggesting that underweight patients may experience increased postoperative complications after radical cystectomy, although no direct link between malnutrition and complications was identified (42). Additionally, evidence indicates that patients diagnosed with clinical T1 bladder cancer, treated with Bacillus Calmette-Guérin immunotherapy or transurethral resection and categorized as obese, have worse cancer-specific outcomes compared to their non-obese counterparts (43, 44). The observation that the obesity paradox diminishes in early-stage cancer hints at the potential root of this phenomenon in bladder cancer, suggesting that the better compliance and fitness of obese patients in withstanding intense treatment and surgical complications may contribute, and these advantages may diminish as treatment becomes less demanding.

The potential underlying mechanism of the obesity paradox, amidst its confounding factors, involves various speculations. The fat mass and obesity-associated protein (FTO), known for its association with body mass and obesity, influences the energy metabolism of cancer cells. However, studies have indicated a significant decrease in FTO mRNA expression in bladder urothelial carcinoma compared to controls, suggesting an oncogenic role in bladder cancer (45). Molecular pathways like PI3K/AKT/mTOR, activated in obese patients due to high circulating levels of IGF1, could be a potential therapeutic target in bladder cancer patients with high BMI (46). The transcription factor Nuclear factor erythroid 2–related factor2 (Nrf2), linked to detoxification and antioxidant response, may correlate with obesity and insulin resistance (47), impacting resistance to cisplatin and overall bladder cancer-specific survival (48). Adipokines released by adipose tissue may also play a role. Kashiwagi et al. (49) demonstrated that the downregulation of adiponectin expression and the upregulation of leptin expression were independent predictors for the recurrence of non-muscle-invasive bladder tumors and the progression of muscle-invasive bladder tumors, respectively. These findings suggest that synthetic adiponectin may exhibit antitumor activity against bladder cancer (49). Furthermore, in bladder cancer patients receiving neoadjuvant platinum-based therapies, low/intermediate mRNA levels of BRCA1 were associated with increased tumor pathological response and overall survival (50), while DNA damage in normal breast epithelia of women with a BRCA mutation positively correlated with BMI and biomarkers of metabolic dysfunction (51).

This study is notable for its comprehensive, population-based approach, including both non-muscle invasive and muscle-invasive bladder cancer in the context of the obesity paradox. However, it has several limitations. Firstly, the method of collecting anthropometric data, such as BMI, is not specified; if self-reported, this may introduce inaccuracies due to recall bias. Direct measurements by healthcare professionals are preferable for accuracy. Secondly, reliance solely on BMI may not fully capture the impact of obesity on bladder cancer outcomes, as it does not account for body fat distribution or other indicators like waist-to-hip ratio (WHR).

In Taiwan, Traditional Chinese Medicine (TCM) is frequently integrated with conventional treatments to support cancer patients. The National Health Insurance program covers TCM, making it accessible to the majority of the population. Many cancer patients use TCM to alleviate the side effects of conventional therapies, manage cancer cachexia (52), enhance quality of life, and potentially improve survival rates through mechanisms such as the regulation of ion channels in cancer cells (53). Consequently, TCM usage could introduce bias if not adequately controlled in studies. For example, TCM use might be more common among individuals with anorexia and cachexia, who may experience better outcomes due to this additional support. This could lead to collider bias and reverse causation, where the association between TCM usage and improved outcomes may reflect the selective use of TCM among patients with specific conditions rather than a direct therapeutic effect.

Additionally, confounding variables such as smoking, socioeconomic status, and diet were not extensively controlled, potentially affecting the observed relationship between BMI and cancer outcomes. Collider stratification bias could also be a concern, as smoking, a risk factor for bladder cancer, may create an artificial inverse association with BMI in non-obese patients. Finally, the follow-up duration may be too short to capture long-term survival outcomes, and the findings may not be generalizable beyond the Taiwanese population to other demographic groups with different obesity profiles and cancer outcomes.

## Conclusion

The complexity of the relationship between BMI and bladder cancer outcomes stems from the varying impacts of BMI across different cancer stages and the observed “obesity paradox.” Previous research has added to this complexity by demonstrating conflicting results that both support and challenge the existence of the obesity paradox. Our study reveals an obesity paradox in which overweight patients exhibit a 0.76-fold reduction in overall mortality and a 0.82-fold reduction in specific mortality compared to normal-weight patients. Conversely, underweight patients show a 1.58-fold increase in overall mortality and a 1.50-fold increase in specific mortality. This study advances existing knowledge by providing a comprehensive analysis of both non-muscle invasive bladder cancer and muscle-invasive bladder cancer within the context of the obesity paradox.

The clinical implications underscore the importance of considering BMI as a prognostic factor in bladder cancer management, with potential benefits of integrating BMI-based assessments into clinical guidelines. Incorporating BMI-specific follow-up strategies—particularly using the WHO Asia-Pacific BMI definitions—may help tailor risk assessments and interventions to regional population characteristics. Further research is warranted to elucidate the mechanisms underlying the obesity paradox in bladder cancer, including the influence of metabolic and inflammatory pathways. Additionally, given the limitations of BMI as an indicator of adiposity, alternative measurements like body composition analysis may provide more precise insights into the impact of adiposity on cancer outcomes.

## Data Availability

The datasets presented in this article are not readily available because the data sources are the Taiwan Nation Health Insurance Database and Taiwan Cancer Registry. The data are available with permission from the Taiwan Health and Welfare Data Science Center (https://dep.mohw.gov.tw/DOS/cp-5119-59201-113.html, accessed on May 02, 2024). Restrictions apply to the availability of these data, which were used under license for this study. Requests to access the datasets should be directed to https://dep.mohw.gov.tw/DOS/cp-5119-59201-113.html.
