# Supplementary material for: Navigating the obesity paradox in bladder cancer prognosis—insights from the Taiwan National Health Insurance System Database
Source: Front Nutr. 2024 Dec 11;11:1433632. doi: 10.3389/fnut.2024.1433632 (PMC11669321; doi:10.3389/fnut.2024.1433632)
Supplement: Supplementary file 1 [file Table_1.DOCX]

**Supplemental Table.** The sensitivity analyses using Taiwan’s BMI classification

|  | Overall mortality | | | | Cancer-specific mortality | | |
| --- | --- | --- | --- | --- | --- | --- | --- |
| Taiwan’s BMI classification | Patients | Death (%) | AHR | p-value | Death (%) | AHR | p-value |
| BMI<18.5 | 571 | 330(57.79) | 1.52(1.36-1.71) | <0.0001 | 206(36.08) | 1.45(1.24-1.68) | <0.0001 |
| 18.5≦BMI<24 | 4560 | 1764(38.68) | Ref. |  | 1089(23.88) | Ref. |  |
| BMI≧24 | 5221 | 1475(28.25) | 0.74(0.69-0.79) | <0.0001 | 961(18.41) | 0.80(0.73-0.87) | <0.0001 |
